# Effect of the new rural cooperative medical system on farmers’ medical service needs and utilization in Ningbo, China

**DOI:** 10.1186/s12913-016-1842-3

**Published:** 2016-10-20

**Authors:** Jianhua Chen, Hai Yu, Hengjin Dong

**Affiliations:** 1 Sir Run Run Shaw Hospital, Zhejiang University School of Medicine, Hangzhou, China; 2 Zhejiang University School of Medicine, Hangzhou, China; 3 Center for Health Policy Studies, Zhejiang University School of Medicine, Hangzhou, China

**Keywords:** Farmer, Medical service needs, Medical service utilization, New Rural Cooperative Medical System

## Abstract

**Background:**

Many countries are developing health mechanisms to pursue the goal of universal coverage. In China, a rural health insurance system entitled New Cooperative Medical System (NCMS) has being developed since 2003. This paper aims to explore the changes in the health service needs and utilization among rural residents in Ningbo, China after the implementation of the new rural cooperative medical system (NCMS), and provide evidence to further improve the strategies of NCMS in China.

**Methods:**

Stratified multistage cluster sampling was used to randomly select 10 villages from 5 townships in Yuyao and Fenghua counties of Ningbo Municipality. Eighty families were selected from each village, and face-to-face interviews were conducted by trained investigators to collect data using questionnaires.

**Results:**

The two-week visiting rate and prevalence of chronic diseases among the farmers included in the study was 25.40 and 22.50 %, respectively, which were higher than the levels in 2003 and 2008. The rate of not visiting the healthcare facility amongst those with illness, and the rate of non- hospitalization amongst those who required it were 32.36 and 0.60 %, respectively, which was lower than the levels in 2003 and 2008. Most of the outpatient visits were to the village clinics, while the hospitalizations were mainly to county hospitals.

**Conclusion:**

NCMS greatly affected the utilization of healthcare services from outpatient clinics and improved the hospitalization rate in county hospitals. Financial difficulties are not the major causes of non-hospitalization and non-visiting any longer. These findings suggest that the NCMS policies alleviated the medical burdens of farmers in a certain degree.

**Electronic supplementary material:**

The online version of this article (doi:10.1186/s12913-016-1842-3) contains supplementary material, which is available to authorized users.

## Background

The ultimate goal of rural health reform and development is to establish a mature health care service system that adapts to the socialist market economy and the developmental level of the Chinese economy, and meets the health demands of people [1]. Health service utilization is the objective index that comprehensively describes the interactions between the people’s health service demands and the supply of the health service resources. Analyses of the health service demands and utilization are key aspects of health service research, and could provide important evidence in helping the development of health-promoting programs and the optimal allocation of the regional health resources [2].

The new rural cooperative medical system (NCMS) is the interdependent system for farmers’ health that is organized, guided, and supported by the government, which raises funds through multiple channels (including individual contributions, village support, and government funding) mainly for comprehensive arrangement for the management of serious disease. Farmers are voluntary joining the NCMS. In October 2002, the Chinese government guided the farmers to join the NCMS actively. In 2009, the Chinese government further improved the health system reform and reassured that NCMS should be the basic medical insurance system for farmers [3]. Zhejiang province is among the 4 pilot provinces selected for the implementation of NCMS by the State Council. NCMS has been implemented in the Zhejiang province since 2003 and covered all the 87 counties till 2006.

The fund raising strategies of the NCMS changed in 2007. In 2003, it was recommended that the municipalities should fund at least 15 Yuan for each farmer every year, while the funds from the counties and towns should together be at least 30 Yuan per farmer every year. The matching money paid by each family should be at least the total of the funds from the municipality, county, and town. Villages could help the farmers to pay their share. While in 2007, the funds from the counties and towns increased from 15 to 25 Yuan/farmer/year, the funds from the farmers and villages changed to 30 Yuan/farmer/year. In 2008, the overall funds for each farmer increased to 140 Yuan/year, of which 20 was from the Ningbo Municipality (for the farmers in mountainous areas, this part was 30 Yuan), 45 from the counties, 25 from the towns, and 50 from the farmers. The funds for each farmer further increased to 300 Yuan/year in 2010, of which 45 was from Ningbo Municipality, 130 from the counties, 25 from towns, and 100 from the farmers. No change was made in 2011 after the last survey.

More and more studies about NCMS have been performed after the application of NCMS polices. Some cross-sectional studies have assessed the effects of the NCMS policies on medical service needs and utilization, and found that the hospitalization rate increased in county hospitals and municipal hospitals increased, while the insurance coverage for medical costs in outpatient clinics also increased [4, 5]. A longitudinal study showed that the NCMS improved the utilization of hospitalization services but not outpatient services by farmers; in addition, financial difficulties were still the major cause affecting the utilization of hospitalization services of farmers [6]. However, that longitudinal study only reflected the effects of NCMS in low-income regions, while the studies in developed coastal regions are still lacking. Ningbo is a region with relatively high income, and the longitudinal study in this area could reflect the effects of NCMS in high-income regions. In addition, the NCMS polices changed for several times since 2003, thus longitudinal study could provide more valuable information.

In the present survey, face-to-face interview was conducted in 2011 to evaluate the effects of NCMS. The data were compared with the results in 2003 and 2008 to investigate health service demands and utilization by the farmers and explore the change in patterns, and thus provide evidence in helping the sustainable development of the NCMS.

## Methods

### A brief introduction of study site

All of the data analyzed in this paper was conducted in the Yuyao and Fenghua counties, which are in Ningbo city and located in the southeast of Zhejiang province. Zhejiang province is among the economically developed provinces in China (The GDP in Zhejiang province was 3231.885 billion in 2011, which was the fourth highest in China [7]). There were 3.27943 million farmers in Zhejiang province in 2011, and their mean net income was 13,017 Yuan/year, which was 1.87-times that of average income of farmers all over the country, and has been the highest among all the provinces in China for 27 continuous years [7]. In the present survey, Yuyao and Fenghua, 2 counties with relatively high economical level (mean income for the farmers was 16,518 Yuan in 2011, which was the fourth among the counties in the province [8]) and large population of farmers (3.6823 million farmers in 2011, which was fourth among the counties in the province [8]).

### Sampling

Data were collected by face-to-face interview using the questionnaire recommended by the Ministry of Health with modifications suggested by the School of Public Health, Zhejiang University. All the investigators were recruited from the medical staff from the selected areas. All the investigators had participated in the previous 2 surveys, they were trained uniformly before the present investigation to ensure the quality. Stratified multistage cluster sampling was conducted to obtain the sample as follows: 5 towns were selected from each of the two counties, 2 villages were selected from each town, and then families were selected from each village. The final sample was from 2000 families in 20 villages of 10 towns in the 2 counties [9]. To analyze the changes, the same villages were selected in the past three surveys. All the subjects included were permanent residents in the selected families. The sampling procedures in the present survey were identical to the ones used in the 4th National Health Services Survey, and had also been widely used in other studies (such as the Basic Health Service Project in Rural Areas and the Evaluation of New Rural Cooperative Medical System).

### Questionnaire

The questionnaire used in the 4th National Health Services Survey (2008 edition) was used in the survey to collect the data from the following aspects: 1) health service needs: including the socioeconomic characteristics, self-assessment of the health status, disease prevalence, disabilities, and health risk factors; 2) health service needs and utilization: including treatment for any disease, degree of satisfaction of demands, reasons for dissatisfaction; utilization of the public health services, maternal and child health care, outpatient and emergency departments, and hospitalization; and the payment of the medical expenditures; 3) medical insurance: including the composition of the medical insurance system, coverage of the medical insurance, range and level of compensation, and the operation of the major insurance systems; and 4) degree of satisfaction of the farmers with the service system, service providing processes, and the coverage and level of the medical insurance. The “illness within two weeks” in the present survey was defined as the self-assessment of presence of a disease based on the health care service aspect, and the subjects with at least one of the following characteristics were considered to be suffering from “illness within two weeks”: 1) felt uncomfortable in the past two weeks and referred to medical for treatment; 2) feel uncomfortable in the past two weeks, and self-prescribe some drugs or adjuvant therapies instead of referring to medical settings; 3) feel uncomfortable in the past two weeks but did not refer to medical settings or self-prescribe any treatments, and stopped working or stayed in bed for at least one day. The “two-week prevalence” was estimated with the “illness within two weeks”, and described as the percentage of the subjects with illness in the previous two weeks. The “two-week visiting rate” was estimated based on the frequency of the subjects visiting a healthcare facility for healthcare services in the previous two weeks due to discomfort, and described as the person-time of seeking medical care within the past two weeks. The “hospitalization rate” was estimated by the hospitalization of the subjects within the past one year, and was described as the person-time of hospitalization in the past one year due to illness.

### Survey processes and quality control

As described, the questionnaire used in the 4th National Health Services Survey (2008 edition) was used for the data collection through face-to-face interview (Additional file 1). The survey was organized by the local health bureau and directed by a group in the Centers for Disease Control and Prevention (CDC) in each selected county. The health bureau of the county was in charge of the training of the investigators and directors, organizing the survey in the county, and the quality control of the survey. Trained investigators were asked to conduct face-to-face interview to collect the data with the questionnaire. The organization, direction, inspection, and quality control of the survey were conducted by the trained directors [9].

### Data process and statistical analyses

Two investigators were asked to input the data with EpiData3.1 software independently. The data were checked and then output into an EXCEL database, and analyzed with SPSS 16.0 software. Chi-square test was used for the analyses. *P* ≤ 0.05 was considered statistically significant.

## Results

A total of 5445 subjects including 2732 males (50.16 %) and 2714 females (49.84 %) were included in the present survey. 70.82 % of them were married, 76 % of them were with the education level of junior middle school or lower.

### Utilization of the health services

#### Health service needs and utilization

Table 1 shows the health service needs and utilization in the past three surveys. The two-week prevalence rate in 2011 was 19.30 %, which was higher than the level in 2003. The non-visiting rate in the ones with diseases within the past two weeks and the non-hospitalization rate in the ones needed to be hospitalized was 32.36 and 0.60 %, respectively. The two-week visiting rate and prevalence of chronic diseases was 25.40 and 22.50 %, respectively. The hospitalization rate in 2011 was 3.40 %, which is similar to the level, compared with 2003 and 2008 (Table 1).

**Table 1:** Comparisons of the health service needs among the 3 surveys

	2003	2008	2011	*χ* ^2^	*P value*
N	4310	4176	5445	-	-
Two-week prevalence (%)	468 (10.86)	852 (20.40)	1051 (19.30)	169.76	<0.001
Two-week visiting rate (%)	493 (11.44)	745 (17.85)	1383 (25.40)	310.69	<0.001
Non-visiting rate in the ones needed visiting (%)	1880 (43.61)	1604 (38.40)	1762 (32.36)	131.35	<0.001
Prevalence of chronic diseases (%)	588 (13.64)	768 (18.38)	1225 (22.50)	125.04	<0.001
Hospitalization rate (%)	129 (2.99)	178 (4.26)	185 (3.40)	10.50	0.005
Non-hospitalization rate in the ones needed hospitalization (%)	981 (22.75)	46 (1.09)	33 (0.60)	2038.95	<0.001

#### Reasons for not visiting or hospitalization

The findings of the present survey showed that 12.20 % of the non-visiting subjects reported financial difficulties, which was lower than in the previous two surveys.46.67 % of the non-visiting subjects felt the diseases were not severe. 26.66 % of the non-visiting subjects reported unavailability of effective treatments (Table 2).

**Table 2:** Causes of non-visiting in the 3 surveys (%)

Year	Felt not severe	Financial difficulties	Unavailable of effective treatments	No time	Traffic inconvenience	Others
2003	52.78	29.17	12.50	0	0	5.55
2008	46.67	26.67	26.66	0	0	0
2011	58.54	12.20	17.07	1.22	0	10.98
*χ* ^2^		112.04				
*P* value		0.00				

33.3 % of the non-hospitalized subjects reported financial difficulties, which was lower than in the previous two surveys, suggesting that financial difficulties was no longer the major cause of non-hospitalization. 48.18 % of the subjects reported feeling not necessary to be hospitalized (Table 3).

**Table 3:** Causes of non-hospitalization in the 3 surveys (%)

Year	Not necessary	Financial difficulties	Unavailable of effective treatments	No time	Poor health services	Others
2003	8.33	83.33	2.78	2.78	2.78	0
2008	10.20	65.12	8.16	0	0	16.52
2011	48.14	33.33	0	7.40	0	11.13
*χ* ^2^	146.88	121.34				
*P* value	0.00	0.00				

#### Selection of medical settings

Village clinics were the major choice for the farmers in all the 3 surveys, and the percentage in 2011 was as high as 86.34 %. However, the percentage of township hospitals and county hospitals was 2.48 and 3.11 % in this study, respectively, which were decreasing gradually in the 3 surveys. The medical services obtained by the subjects were mainly from the county hospitals (81.10 % in the present survey), followed by the hospital above the county level (11.50 % in the present survey). However, the services obtained from the township hospitals decreased gradually with year (7.40 % in the present survey; Table 4).

**Table 4:** Percentages of visiting and hospitalization in the hospitals with different levels (%)

Percent (%)			
	2003	2008	2011
Two-week visiting^a^			
Village clinics	46.40	72.41	86.34
Township hospitals	24.10	6.90	2.48
County hospitals	22.66	14.42	3.11
Hospitals above county level	6.84	6.27	8.07
Hospitalization within 1 year^b^			
Village clinics	-	-	-
Township hospitals	22.50	15.60	7.40
County hospitals	54.17	58.50	81.10
Hospitals above county level	23.33	25.90	11.50

-: not available

Two-week visiting^a^ *χ* ^2^ = 48.86 *P* = 0.002

Hospitalization within 1 year^b^ *χ* ^2^ = 116.32 *P* = 0.00

## Discussion

The two-week prevalence of the farmers was 19.30 % in the present survey, which was similar to the level in the selected areas but higher than the national level in 2008. The two-week visiting rate showed an incremental increase in the 3 surveys and reached 25.40 % in the present survey, which was also higher than the national level in 2008 (15.2 %) and 2003 (13.92 %). The increased two-week visiting rate was associated with the increased insurance coverage for medical costs in outpatient clinics. The hospitalization rate was 3.40 % in the present survey, which was lower than the national level in 2008 (6.8 %). However, the non-hospitalization rate in the ones needed hospitalization was 0.6 % in the present survey, which was significantly lower than the level in the selected areas in both 2003 and 2008, as well as the national level in 2008 (24.7 %) [10, 11]. These findings suggest that regarding the compensation strategy in Ningbo (after paying the share, the participants are covered by the medical insurance from January 1 to December 31 in the next year, and could be compensated for hospitalization, outpatient visiting for specific diseases, and ailments, as well as free physical examinations for 2 years. For the families that the parents participated, the newborns could also participate at any time. For the newborns that participated within 3 months after the birth, the medical expenditures after the birth is also covered by the NCMS.), the major effectiveness are about satisfying the demands of hospitalization of the farmers. Comparing with the national level, the non-hospitalization rate in the ones needed to be hospitalized was substantially lower in Ningbo after the implement of NCMS, which was in consistence with the findings in Wuyuan county reported by Yuan et al. [12].

Our survey showed that financial difficulties were not the major causes of not visiting healthcare facility or getting hospitalized in the farmers in Ningbo any longer. The percentage of financial difficulties decreased gradually in the 3 surveys, suggesting that the low income was not the major cause. The findings were not consistent with the results reported by Yuan et al. [12], which could be associated with the difference in the financial level between the farmers in Ningbo and Wuyuan. Similarly, the major cause of not hospitalization also changed from financial difficulties to feeling not necessary. These findings suggest that the NCMS effectively reduced the financial burdens of the farmers.

Visiting rate in different medical settings reflects the demands for different levels of health services. Village clinics were the major choice for the farmers, which was increasing as comparing with the previous two surveys. The percentage of two-week visiting was 86.34 % for the village clinics in the present survey, which could be associated with the insurance coverage for medical costs in outpatient clinics; in addition, the insurance coverage in village clinics was also evidently higher than in county hospitals. Furthermore, the rapid development of the village clinics in the recent years could also play a role. The village clinics were very convenient for the farmers and could effectively cure common diseases, thus more and more farmers tended to seek health care from village clinics. Therefore, the village clinics should be further developed and the clinicians should be further trained to improve their skills, which will better serve the farmers with economical and convenient health care services.

The results of the 3 surveys showed that the percentage of hospitalization in township hospitals was decreasing, suggesting that compensatory strategy of making township hospitals priority (the threshold for compensation was down-regulated in 2010, and the compensation proportion was adjusted to 65 % for hospitalization in township hospitals, 55 % in county hospitals, and 55 % in hospitals above county level [13]) only resulted in limited effectiveness. Farmers did not come to township hospital more often for the increased compensation proportion; instead, they tended to select hospitals based on the health service quality and level. Therefore, we believe that further strengthening the training of medical staff and constructing the infrastructures of township hospitals to improve the service qualities and levels in addition to increase the differences of compensation proportion among the hospitals with different levels could guide more farmers seeking health care in primary hospitals, which not only improves the convenience but also decreases the medical expenditures and reduces the risk of the using the funds of NCMS.

The utilization of health services by hospitalization was still dominant by county hospitals, and the percentage kept increasing and reached 81.10 % in the present survey, suggesting that the county hospitals are still the major choice for farmers to obtain health services. With the increased insurance coverage in NCMS, more farmers tended to seek medical services in county hospital and higher hospitals. Therefore, improving the infrastructure of county hospitals to increase the service qualities and levels could meet the hospitalization demands of the farmers. Strengthening the management of medical expenditures in the hospitals of different levels could also help managing the hospitalization cost, reducing the burdens of the farmers, and increasing the capability of NCMS in coping with risks.

## Conclusion

NCMS greatly affected the utilization of healthcare services from outpatient clinics and improved the hospitalization rate in county hospitals. The NCMS policies alleviated the medical burdens of farmers in a certain degree. The results of this study should be interpreted with cautions. First, the data only contains a short time (two weeks) of information about formal service; second, only two counties are conducted, both of which are among the high-income regions, thus the results could not reflect the situation in China; and finally, the effects of the differences between these two counties, as well as the differences in the income of the farmers on the NCMS are not investigated. All of the issues above calls for future study.

## Additional file

Additional file 1: Questionnaire of the Health Services Survey. (DOCX 19 kb)

## Abbreviation

NCMS: New rural cooperative medical system
