# Supplementary material for: Effect of the new rural cooperative medical system on farmers’ medical service needs and utilization in Ningbo, China
Source: BMC Health Serv Res. 2016 Oct 20;16:593. doi: 10.1186/s12913-016-1842-3 (PMC5072316; doi:10.1186/s12913-016-1842-3)
Supplement: Additional file 1: — Questionnaire of the Health Services Survey. (DOCX 19 kb) [file 12913_2016_1842_MOESM1_ESM.docx]

**Explanation for the “questionnaire”**

The whole household health questionnaire used in the National Health Service Survey (NHSS) includes 8 instruments and in this study we just used part of them. So, the related questions and answers were listed and translated in English as below:

**一．家庭一般情况问卷Instrument for Characteristics of The Family:**

1.您家年收入是多少（元）？（纯收入）

How much was your annual household income(net income)?

2.您家是否参加了新型农村合作医疗？

Did you take part in the New Rural Cooperative Medical System?

**二．家庭成员健康调查问卷Instrument for Characteristics of The Family Members:**

1.性别 男 女

Sex male female

2.出生日期 （年） （月）

Date of birth (year) (month)

3.婚姻状况 未婚 在婚 离婚 丧偶 其他

Marital status: (1) unmarried (2) currently married (3) divorced (4) widowed  (5)or other

4.文化程度 没上过学 小学 初中 高中/技校 中专/中技 大专 大学及以上

Educational level: (1)Haven't been to school (2)primary school (3)junior middle school (4)high school/ technical school (5)secondary technical school (6)junior college (7)university and above

5.就业状况 在业 离退休 在校学生 无业或失业

The employment situation: (1)employed (2) retired  (3)student in the school (4)unemployed

B 患病及受伤情况

6.调查前14天内，您是否有身体不适? ⑴是 ⑵否

How are you in recent 2 weeks? Yes no

7.半年内您是否患有**经医生诊断**的慢性疾病? ⑴是 ⑵否

Do you have any chronic disease in recent half a year? Yes no

1. 一种疾病 （疾病名称）

Name of the disease

1. 查填第一种疾病编码

Code of the disease

1. 确诊时间： ⑴半年前 ⑵半年内

Diagnose time half a year ago in recent half a year

1. 半年内是否进行了治疗？ ⑴是 ⑵否

Treatment in recent half a year? Yes no

8.过去12个月内，您是否因病伤、体检、分娩等原因住过医院？ ⑴是 ⑵否

Have you been in hospital for disease or health physical examination in recent 12months? Yes no

9.过去12个月内，是否有医生诊断您需要住院，而您没住的情况？ ⑴是 ⑵否

Do you have the experience that you refuse the doctor’s advice which you should be in hospital in recent 12 months yes no

10.最近这次未住院的原因**：** ⑴没必要 ⑵无有效措施 ⑶经济困难

(4)医院服务差 ⑸无时间 ⑹无床位 ⑺其它

The reason for not being hospitalized : (1)not necessary (2) no effective measure (3) financial difficulties (4)hospital bad service (5)no enough time (6)no bed (7)other

11.近2周内最主要的不适是什么？ ⑴发烧 ⑵疼痛 ⑶腹泻 ⑷咳嗽

1. 心慌/心悸 ⑹其它 ⑺无自觉症状

What symptom do you feel in previous 2 weeks? (1)fever (2)pain(3)diarrhea

(4)cough (5)palpitation (6)other (7)no symptom

12.您患病后，是否进行了治疗（包括自我医疗）？ ⑴是 (2)否

Have you taken the treatment ? yes no

13.如未治疗，最主要的原因是什么？ ⑴自感病轻 ⑵经济困难 ⑶无时间

⑷交通不便 ⑸无有效措施 ⑹其它

If you doesn’t receive treatment, which is the main reason?

(1)self-belief of slight illness (2)financial difficulties(3)no time (4)traffic inconvenience (5)no effective measure (6)other

14.第一次就诊是在哪里？

⑴私人诊所 ⑵卫生室/站 ⑶乡镇街道卫生院/社区卫生服务中心 ⑷县/市/区医院 (5)县/市/区中医医院 ⑹市/地医院 ⑺市/地中医院 ⑻省级医院 ⑼省中医院 ⑽其它

Where do you go to see the doctor?

(1)village clinics (2)community health station(3) community health center /township hospitals(4)county-level hospitals (5) county-level Chinese traditional hospitals (6)municipal-level hospitals (7) municipal-level Chinese traditional hospitals (8)provincial hospitals (9)provincial Chinese traditional hospitals (10)other

15.您是在下列的哪类医疗机构住院的？

⑴乡镇街道卫生院/卫生服务中心 ⑵县/市/区医院 ⑶县/市/区中医医院 ⑷市/地医院　　　⑸市/地中医院 ⑹省级医院 ⑺省中医院 ⑻其它

Hospitalization site :

(1)community health center (2)county-level hospitals (3) county-level Chinese traditional hospitals (4)municipal-level hospitals (5) municipal-level Chinese traditional hospitals (6)provincial hospitals (7)provincial Chinese traditional hospitals (8)other
